# Promoting Colonization in Metastatic HCC Cells by Modulation of Autophagy

**DOI:** 10.1371/journal.pone.0074407

**Published:** 2013-09-13

**Authors:** Yuan-Fei Peng, Ying-Hong Shi, Ying-Hao Shen, Zhen-Bin Ding, Ai-Wu Ke, Jian Zhou, Shuang-Jian Qiu, Jia Fan

**Affiliations:** 1 Department of Liver Surgery, Liver Cancer Institute, Zhongshan Hospital, Fudan University, Key Laboratory of Carcinogenesis and Cancer Invasion of Ministry of Education, Shanghai, China; 2 Institutes of Biomedical Sciences, Fudan University, Shanghai, People’s Republic of China; University of Modena & Reggio Emilia, Italy

## Abstract

**Background:**

Autophagy is an important adaptive survival mechanism, which has been postulated to be involved in cancer metastasis. The purpose of this study was to investigate autophagy in metastasis of hepatocellular carcinoma (HCC).

**Methods:**

Immunohistochemical analysis of autophagic activity in metastatic and paired primary HCC tissues using LC3 as autophagosome marker was performed in samples from 216 HCC patients diagnosed with metastasis (including 158 intravascular, 42 intrabiliary, 8 lymph node, 4 bone and 4 lung metastases). Then a mouse model of pulmonary metastasis was established using a highly metastatic HCC cell line (HCCLM3). Autophagy in pulmonary metastases and paired primary tumors were analyzed by LC3 immunohistochemistry, transmission electron microscopy (TEM) and western blot analysis. Further, mouse model of pulmonary metastasis and *in* *vitro* cell migration, invasion and detachment models were established using a stable GFP-LC3-expressing HCCLM3 cell line (HCCLM3-GFP-LC3). Autophagic alterations during metastatic colonization, migration, invasion and detachment were determined by GFP-LC3 analysis and western blot analysis.

**Results:**

LC3 immunohistochemistry of metastases and primary tumors from HCC patients revealed significantly higher LC3 expression in metastases than primary HCC, which suggested a higher level of autophagy in HCC metastases. Further immunohistochemical, TEM, western blot and *in* *vivo* GFP-LC3 analyses of lung metastases and primary tumors in mouse model of pulmonary metastasis confirmed that metastatic colonies displayed higher level of autophagy than primary tumors and the early metastatic colonies displayed highest level. The dynamic monitoring of autophagy in cell migration, invasion and detachment showed that autophagy did not significantly alter in those processes.

**Conclusions:**

Autophagy is activated in metastatic colonization but not in invasion, migration and detachment of HCC cells. Autophagy may play a role in HCC metastasis via promoting metastatic colonization of HCC cells.

## Introduction

Autophagy is a self-degradative process by which cells break down cytoplasmic materials in the lysosome. It serves as a dynamic recycling system that produces new building blocks and energy for cellular homeostasis and renovation [1]. As a cytoprotective survival pathway, it confers stress tolerance, limits damage and sustains viability under adverse conditions [1-5]. It has been demonstrated that autophagy can protect cancer cells against hypoxia, metabolic stress, detachment-induced anoikis and diverse cellular damages, as well as apoptosis or necrosis induced by anti-tumor therapy or other cell death stimuli [2,5-12].

Metastasis is the major cause of death from cancer, which has been linked to cell death resistance [13,14]. As an important pro-survival mechanism autophagy has been postulated to play a role in cancer metastasis [11]. It is hypothesized that autophagy may be activated during metastasis and be exploited by metastatic cancer cells to adapt and survive unfavorable stresses conditions. For example, autophagy may be activated to function as an alternative energy source to overcome metabolic stress which is often faced by metastatic tumor cells, especially tumor cells that metastasize to organs that provide a poor supply of nutrients [15]. It may also be activated after cell detachment from the extracellular matrix (ECM) to resist anoikis induction and sustain cell survival as metastatic cancer cells disseminate in the circulatory system without proper cell-ECM contact [16].

However, autophagy in HCC metastasis remains unknown. This is largely due to technical difficulties in analyzing autophagy in metastasis. Traditional ultrastructural analysis using transmission electron microscopy (TEM) is standard technique for analyzing autophagy. However, it has many limitations and is often difficult to perform *in vivo* analysis of autophagy (especially quantitative analysis and dynamic observation), which is not suitable for analysis of autophagy in metastasis. In recent years, the immunohistochemical analysis using microtuble-associated protein light chain 3 (LC3) as autophagosome marker emerges as a valuable technique for *in vivo* analysis of autophagy (especially in situ detection of autophagy) [17-19]. Meanwhile, *in vivo* GFP-LC3 analysis was also reported to be a useful approach for *in vivo* autophagy assay [20]. And it was shown to be able to compensate the disadvantages of LC3 immunohistochemical analysis as assaying autophagy in tissue using LC3 as autophagic marker appears to be valuable only when LC3 protein is overexpressed [21-23].

In this study, we combined LC3 immunohistochemical analysis, *in vivo* GFP-LC3 assay, western blot and TEM analysis to examine autophagy in HCC metastasis and determine the potential role of autophagy in HCC metastasis. Specifically, a comparative LC3 immunohistochemical analysis of metastatic and primary HCC tissues was performed in samples from HCC patients with metastasis. Then a mouse model of pulmonary HCC metastasis was established. Autophagy in pulmonary metastases and primary tumors were analyzed by LC3 immunohistochemistry, western blot analysis and TEM. Further, a highly metastatic HCC cell line stably expressing GFP-LC3 reporter was established. Mouse model of pulmonary metastasis and *in vitro* cell migration, invasion and detachment models were developed using the GFP-LC3-expressing HCC cells. Autophagic alterations during metastatic colonization, migration, invasion and detachment were determined.

## Materials and Methods

### Ethics Statement

*Ethics Statement of Human sample*: Ethical approval was obtained from the Research Ethics Committee of Zhongshan Hospital, Fudan University, and written informed consent was obtained from each patient. *Ethics Statement of Animal*: All animals were cared for and handled according to the Guide for the Care and Use of Laboratory Animals published by the National Institutes of Health. Experimental protocol was approved by Shanghai Medical Experimental Animal Care Committee.

### Cell lines and Animals

Human HCC cell line with high metastatic potential established by our institute (HCCLM3) was routinely maintained [24,25]. Male BALB/c nu/nu mice (6 weeks old, Chinese Academy of Science) were bred in specific pathogen-free conditions. All of the mice were cared for and handled according to the Guide for the Care and Use of Laboratory Animals published by the National Institutes of Health.

### Immunohistochemical analysis

Paired metastatic and primary HCC samples from patients or mouse model of pulmonary metastasis were analyzed by immunohistochemistry using LC3 as autophagosome marker. The patient samples were obtained with informed consent from 216 HCC patients who were diagnosed with metastasis in Zhongshan Hospital of Fudan University between 2004 and 2008. The samples consist of 200 intrahepatic metastases (158 intravascular and 42 intrabiliary metastatic tumor emboli) and 16 extrahepatic metastases (8 porta hepatis lymph node, 4 bone and 4 lung metastases). Demographic and clinical characteristics of the patients are summarized in Table 1. The intrahepatic metastases samples were constructed into tissue microarrays (TMAs) and immunohistochemically analyzed using LC3B antibody (1:50, Cell Signaling), while the extrahepatic metastases were directly sectioned and immunohistochemically analyzed using LC3B antibody (1:100, Cell Signaling). The murine samples (6 paired pulmonary metastases and primary tumors) were obtained from mouse model of pulmonary metastasis which was established using HCCLM3 cells as described below. The samples were immunohistochemically analyzed using LC3B antibodies (1:100, Cell Signaling). The LC3 staining was analyzed using computer-aided and pathologist visual scoring. The images were obtained using Axioplan microscope (Zeiss). Ten randomly selected discontinuous fields per section at ×200 were evaluated blindly. Image-Pro Plus 6 software was used to quantify the integrated optical density (IOD). Comparisons of the IOD were made by 2-tailed paired samples *t*-test (n>10) or Wilcoxon signed-rank test (n<10) between two groups.

**Table 1:** Demographic and Clinical Characteristics of 216 HCC Patients with metastasis.

	Intravascular metastasis	Intrabiliary metastasis	Lymph node metastases	Bone metastases	Lung metastases
Number of patients	158	42	8	4	4
Sex (Female/male)	79/79	20/22	4/4	2/2	2/2
Median age (yr)	49.5	52	57.5	49.5	47
Preoperative Chemotherapy	No	No	No	No	No
Hepatitis B virus infection, %	100 (158/158)	100 (42/42)	100 (8/8)	100 (4/4)	100 (4/4)
Liver cirrhosis, %	60 (95/158)	64 (27/42)	36 (3/8)	25 (1/4)	25 (1/4)

### Generation of HCC cells stably expressing GFP-LC3 and fluorescent microscopic analysis

A highly metastatic HCC cell line stably expressing GFP-LC3 was generated using lentivirus-mediated GFP-LC3 overexpression. A lentiviral vector containing GFP-LC3 reporter (pLV-puro-GFP-LC3) was constructed. HCCLM3 cells were transfected with lentivirus particles (MOI=20) and then selected in media containing 2µg/ml puromycin. The stable GFP-LC3-expressing HCCLM3 cells were termed as HCCLM3-GFP-LC3. A stable GFP-expressing HCCLM3 cell line (HCCLM3-GFP) mediated by pLV-puro-GFP lentivirus served as control. The cells were observed under fluorescent microscope. The number of GFP-LC3 dots per cell was determined using Top-Hat algorithm of Image-Pro plus 6 (MediaCybernetics) and manually counting. For all of the experiments displayed, fresh transfectants were used. Each experiment was performed with cells derived from the same origin. The number of passages of the cells used in each group was the same.

### Mouse model of pulmonary metastasis

A well-established nude mouse model of pulmonary metastasis was used [24]. The mouse model of pulmonary metastasis was established as previously described [26]. Briefly, 3.0×10^6^ HCCLM3 or HCCLM3-GFP-LC3 cells were suspended in 100µl serum-free DMEM and Matrigel (BD) (1:1) and then inoculated into the liver parenchyma of nude mice under anesthesia with ketamine after opening up the abdomen. The mice were monitored once every three days. The animals were sacrificed six weeks later. The lungs and livers of the mice were resected for further analyses.

### Electron microscopy

Mouse model of pulmonary metastasis was established using HCCLM3 cells as mentioned above. The mice (n=6) were killed six weeks later. The livers and lungs were resected and fixed in glutaraldehyde (3%) at 4°C for 6 h. Then the specimens were washed in PBS and post-fixed with 1% OsO4 for 2 h. After dehydration in graded alcohols, the specimens were embedded in Epon 618 (Ladd Research). Ultrathin sections were stained with lead citrate and uranyl acetate and examined through CM-120 transmission electron microscope (Philips). For each sample 50 sections were made and the number of autophagic vacuoles per cell in each section was counted.

### Fluorescence microscopy of GFP-LC3-expressing tissues

Mouse model of pulmonary metastasis was established using HCCLM3-GFP-LC3 cells as mentioned above. The mice (n=6) were killed six weeks later. The livers and lungs were resected immediately and embedded in OCT (Tissue-Tek) at -20°C. The samples were sectioned at 40µm thickness with cryostat (Leica). The sections were stained with DAPI (1:1000 dilution, 15min, Invitrogen) and observed under fluorescence microscope (Leica). Multichannel images were taken and analyzed with Image-Pro plus 6 (MediaCybernetics). Autophagy in GFP-LC3 expressing metastatic and primary tumor tissues was quantified by counting the number of GFP-LC3 dots divided by GFP-LC3-expressing area (number of GFP-LC3 dots per area).

### Assaying autophagy in cell migration

*In vitro* migration model was established using transwell and HCCLM3-GFP-LC3 cells. Briefly, 2×10^5^ cells were suspended in 200µl DMEM with 1% BSA and seeded on the top chamber of the transparent 8µm pore polycarbonate transwell (Millicell). Full medium (900µl, DMEM with 10% FBS and NIH3T3 supernatant) was added to the bottom chamber. The transwell with cells was placed in live cell imaging station (PerkinElmer) and the cells were allowed to migrate for 12h. The alterations of autophagic activity during cell migration were dynamically monitored under confocal microscopy (Olympus) and analyzed using volocity software (PerkinElmer). The number of GFP-LC3 dots per cell in migrated and non-migrated cells was determined using Top-Hat algorithm of Image-Pro plus 6 and manually counting.

### Assaying autophagy in cell invasion

*In vitro* invasion model was established using matrigel invasion assay with HCCLM3-GFP-LC3 cells. Briefly, 2×10^5^ cells were suspended in 200µl DMEM with 1% BSA and loaded onto the upper compartment of the transparent 8µm pore polycarbonate transwell (Millicell) that coated with matrigel (BD). The lower compartment was filled with full medium (900µl, DMEM with 10% FBS and NIH3T3 supernatant). The transwell with cells were placed in live cell imaging station (PerkinElmer) and the cells were allowed to invade for 72h. Alterations of autophagic activity during invasion were dynamically monitored. The number of GFP-LC3 dots per cell in invaded and non-invaded cells was determined using Top-Hat algorithm of Image-Pro plus 6 and manually counting.

### Assaying autophagy in cell detachment

*In vitro* cell detachment model was established using culture dishes with ultra-low attachment surface. The cells cultured in the dishes with ultra-low attachment surface were kept in a stationary suspended (unattached) state, which simulated the process that HCC cells detached from extracellular matrix (ECM) and disseminated in the circulation system. The cells growing attached in dishes with standard tissue culture surface served as control. Briefly, 5×10^6^ HCCLM3-GFP-LC3 cells were cultured on dishes with either ultra-low attachment surface or standard tissue culture surface (Corning) and placed in live cell imaging station (PerkinElmer) for 72 hours. The cells were dynamically monitored under confocal microscopy (Olympus) and analyzed by volocity software (PerkinElmer).

### Western blot analysis

Protein extraction of formalin-fixed, paraffin-embedded tissues was performed using Qproteome FFPE Tissue Kit (Qiagen) while protein extraction of the fresh tissues was performed using Qproteome Mammalian Protein Prep Kit (Qiagen) according to the manufacturer’s instructions. Western blot analysis was performed as previously described [27]. The following antibodies were used: anti-human antibodies against LC3B (1:500, Cell Signaling), p62 (1:500, Cell Signaling) or GAPDH (1:5000, Millipore). The protein bands were quantitatively analyzed by Quantity One (Bio-Rad).

### Statistical Analysis

Statistical analysis was performed using SPSS 13.0 software. Values are expressed as mean ± SD of three independent experiments unless stated otherwise. Comparisons of the quantitative data were made using 2-tailed paired samples Student’s *t* test or Wilcoxon signed-rank test between two groups or by one-way ANOVA for multiple groups unless stated otherwise. Statistical significance was set at *P*<0.05. 

## Results

### Autophagy is upregulated in metastases of HCC

**Figure 1:**
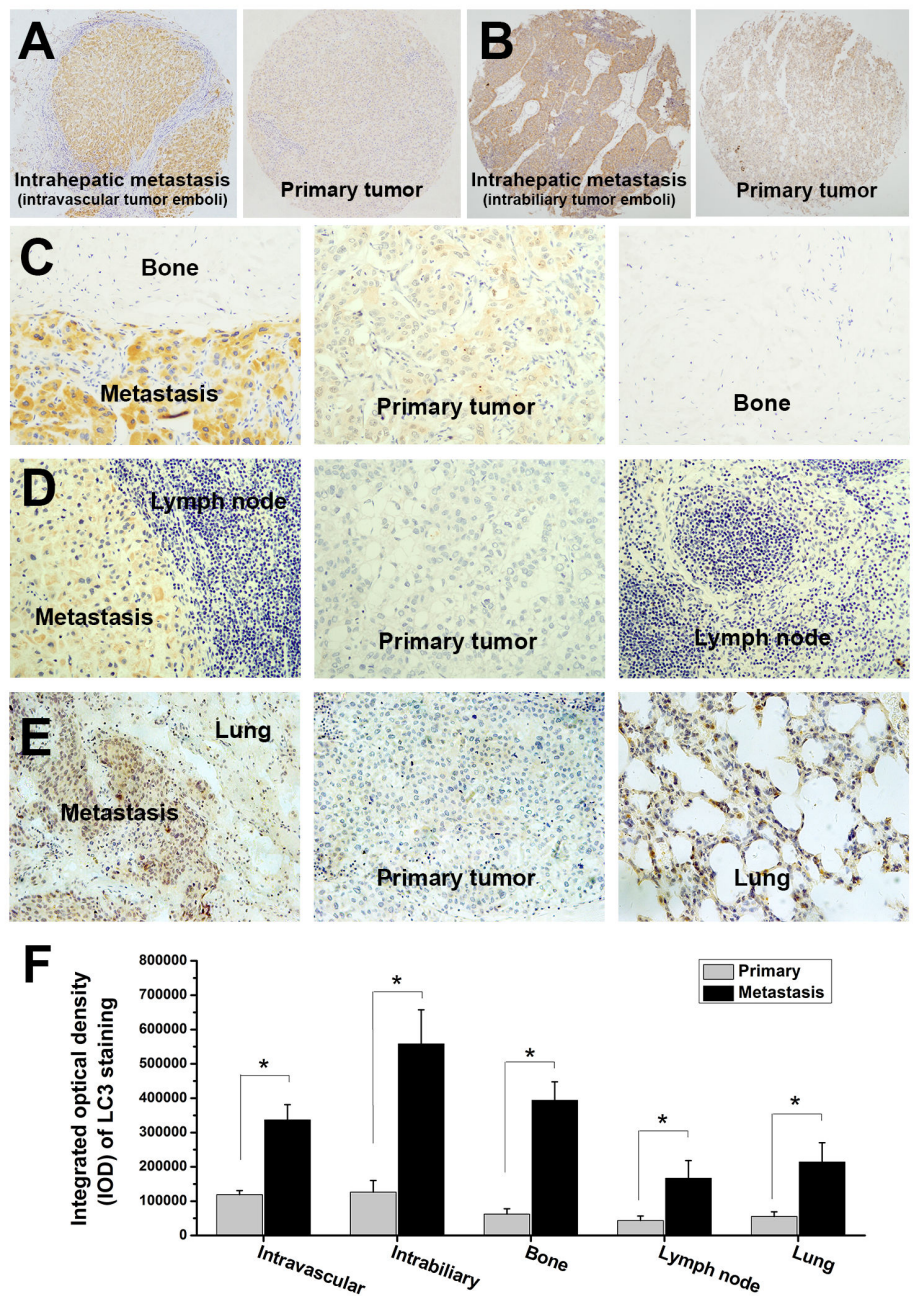
Immunohistochemical analysis of paired metastatic and primary tissues from HCC patients diagnosed with metastasis revealed significantly higher LC3 expressions in metastases than primary tumors. (A, B) TMA analysis of LC3 expressions in intravascular tumor emboli (A), intrabiliary tumor emboli (B) and paired primary tumors. (C, D, E) LC3 expressions in bone metastasis (C), lymphatic node metastasis (D), lung metastasis (E), paired primary tumors and normal tissues. (F) Integrated optical density (IOD) quantification of LC3 expressions in the metastases and primary tumors.

To explore the possible role of autophagy in metastasis, we examined whether autophagy was activated in metastasis. We compared the autophagic activities in metastasis and primary tumor through a combination of LC3 immunohistochemical analysis, transmission electron microscopy (TEM), western blot analysis and *in vivo* GFP-LC3 analysis. Immunohistochemical analysis of primary and metastatic HCC samples from HCC patients using LC3 as autophagosome marker showed that LC3 expressions in metastases were significantly higher than those in primary HCC tissues (Figure 1). The integrated optical density (IOD) of LC3 staining in intravascular metastases, intrabiliary metastases, bone metastases, lymph node metastases and lung metastases were 336680±44698, 558220±99102, 394083±53541, 166563±51412 and 214122±56033, which were significantly higher than those in paired primary HCC tumors (118833±12056, 126490±33543, 62472±15257, 43763±12761 and 55144±13662, respectively) (all *P*<0.001) (Figure 1). The increased LC3 expression in metastases suggested that autophagy was upregulated in metastases. Further western blot analysis of LC3 and p62 showed significant increase of LC3II/LC3I ratio and remarkable decrease of p62 expression in metastases, which indicated that autophagy was upregulated in metastasis of HCC (see Figure S1).

**Figure 2:**
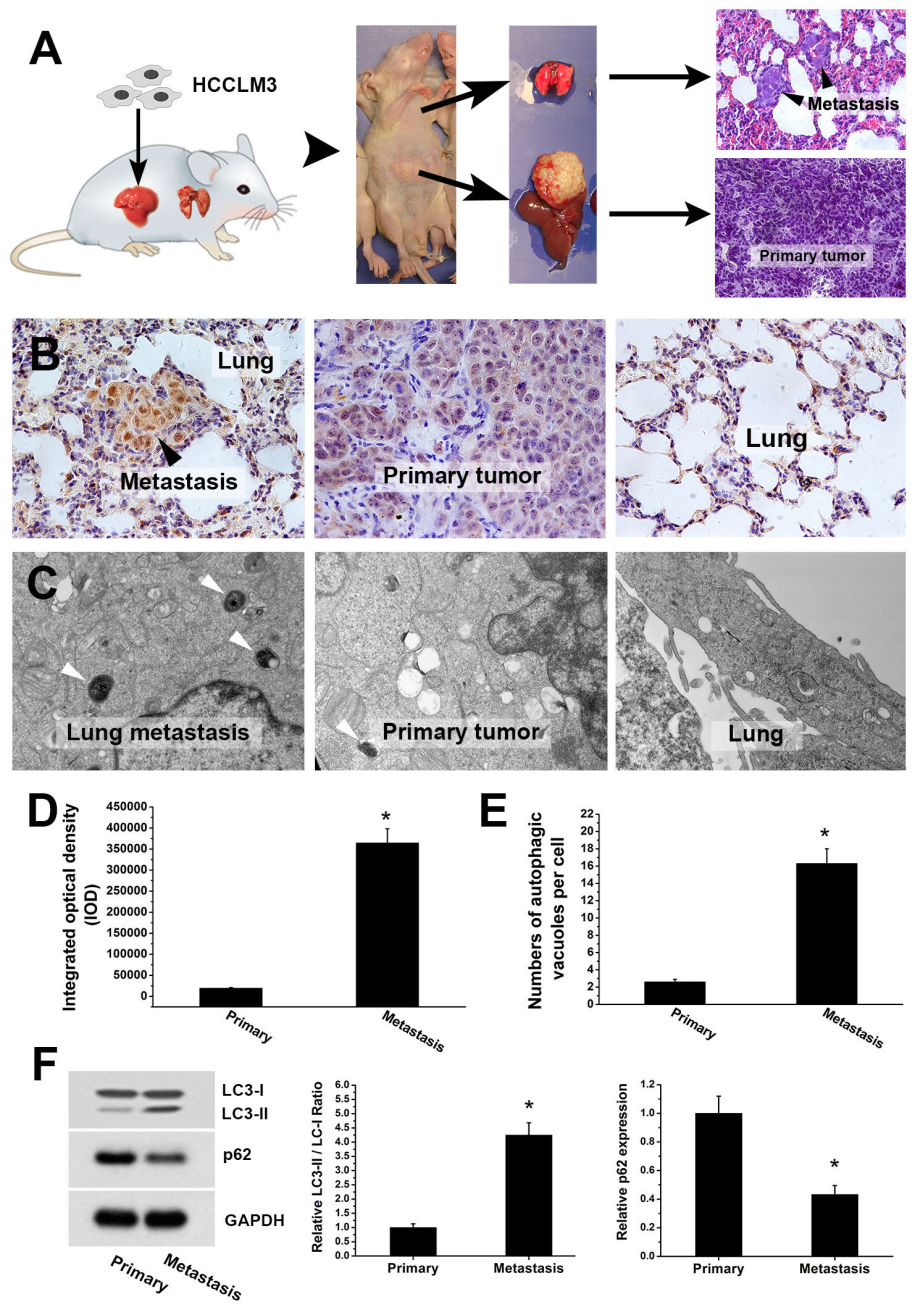
Immunohistochemical analysis, transmission electron microscopy and western blot analysis of lung metastases and paired primary HCC tumors from mouse model of pulmonary metastasis. (A) Establishment of mouse model of pulmonary metastasis using highly metastatic HCC cell line (HCCLM3). (B, D) Immunohistochemical analysis of LC3 expressions in lung metastases and paired primary tumors showed higher LC3 expression in lung metastases. (C, E) Transmission electron microscopic analysis of lung metastases and paired primary HCC tumors revealed increased autophagic vacuoles in lung metastases. White arrows point to autolysosome and autophagosome. (F) Western blot analysis showed significantly increased LC3II/LC3I ratio and remarkably decreased p62 level in lung metastases (*P<0.05).

**Figure 3:**
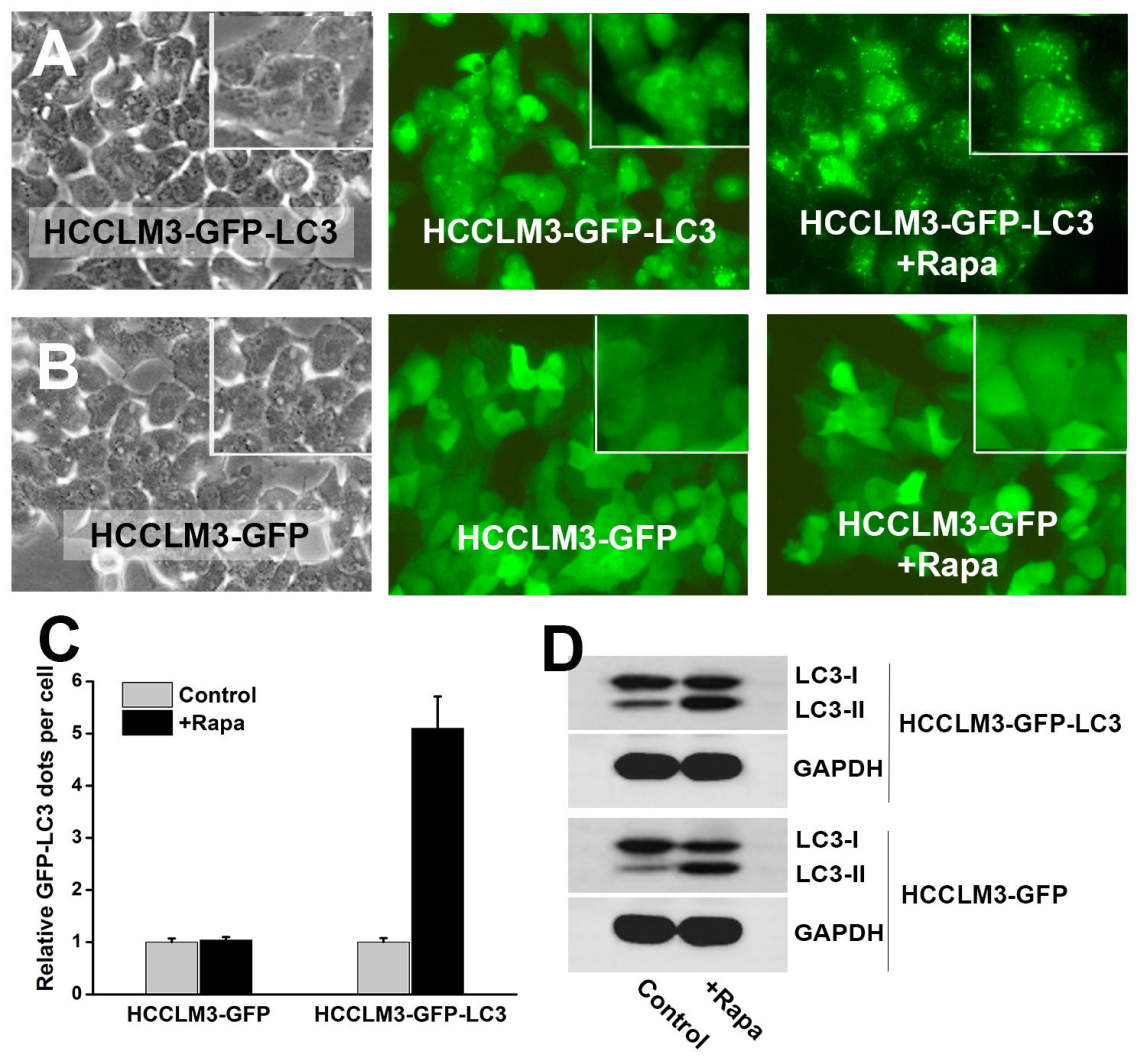
Generation of stable GFP-LC3 expressing HCC cell line. (A, C) Highly metastatic HCCLM3 cell line stably expressing GFP-LC3 reporter gene (HCCLM3-GFP-LC3) was generated via lentivirus-mediated GFP-LC3 overexpression. The HCCLM3-GFP-LC3 cells exhibited a large number of GFP-LC3 dots after rapamycin treatment (+Rapa). (B, C) HCCLM3 stably expressing GFP (HCCLM3-GFP) served as Control. The HCCLM3-GFP cells displayed no GFP-LC3 dots after rapamycin treatment (+Rapa). (D) Western blot analysis of LC3 showed that rapamycin induced intense autophagy in both HCCLM3-GFP-LC3 and HCCLM3-GFP.

**Figure 4:**
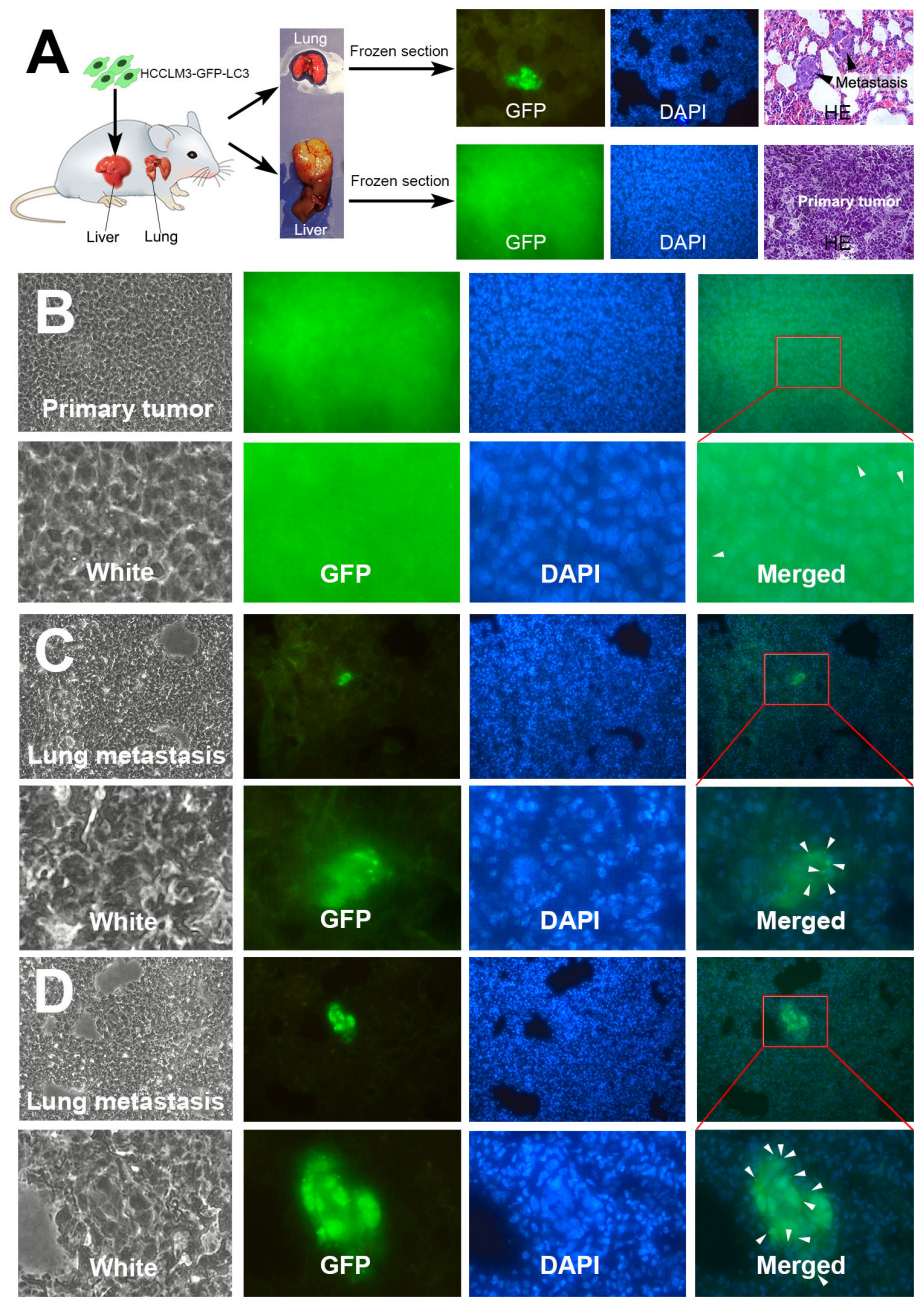
*In vivo* GFP-LC3 analysis revealed higher level of autophagy in metastatic colony as compared with primary tumor. (A) Establishment of mouse model of pulmonary metastasis using HCCLM3-GFP-LC3 cells and fluorescence microscopic analysis of GFP-LC3-expressing lung metastasis and primary tumor. (B) Primary HCC tumors showed low level of autophagy. (C, D) Pulmonary metastatic colonies displayed significantly higher level of autophagy than primary tumors. And early metastatic colonies (C) displayed higher autophagic activity than late-stage metastatic foci (D). Autophagy was quantified by measuring the number of GFP-LC3 dots / GFP-LC3-expressing area. White arrows indicate GFP-LC3 dots.

Due to the limitation of human formalin-embedded sample and potential misinterpretation, we further validated the findings in a well-established mouse model of pulmonary metastasis (Figure 2A). The fresh tissues of pulmonary metastases and primary HCC were analyzed by LC3 immunohistochemistry, TEM, western blot analysis and *in vivo* GFP-LC3 analysis. The LC3 immunohistochemistry of lung metastasis and primary HCC tumors confirmed that LC3 expression significantly increased in metastasis (IOD of lung metastases and primary HCC tumors were 364701±33647 and 19431±1434, *P*<0.001) (Figure 2B,D). TEM analysis showed increased autophagosome in metastasis as compared with primary tumor (Figure 2C,E). The numbers of autophagic vacuoles per cell in lung metastases (16.3±1.7) were significantly higher than that in primary tumors (2.6±0.3) (*P*<0.001). The higher LC3 expression and increased autophagosome in metastatic foci suggested a higher level of autophagy in metastasis of HCC. To avoid the disadvantages of LC3 immunohistochemical analysis (vulnerable to be false positive) and TEM analysis (difficult to quantify), further western blot analysis and *in vivo* GFP-LC3 analysis were performed to validate the findings. Western blot analysis of LC3 and p62 showed significantly increased LC3II/LC3I ratio and remarkably decreased p62 level in lung metastases (Figure 2F). Further, a highly metastatic HCC cell line stably expressing GFP-LC3 (HCCLM3-GFP-LC3) was established via lentivirus-mediated GFP-LC3 overexpression (Figure 3). Mouse model of pulmonary metastasis was then established using the HCCLM3-GFP-LC3 cells (Figure 4A). Fluorescent microscopic analysis of the GFP-LC3-expressing metastatic and primary tumor tissues showed that the number of GFP-LC3 dots per area in lung metastases were significantly higher than that in primary HCC tumor (44.31±4.2 vs. 1.95±0.26, *P*<0.001) (Figure 4B,C,D). The western blot and GFP-LC3 analyses confirmed the findings from LC3 immunohistochemistry and TEM. The upregulated autophagy in metastasis of HCC suggests that autophagy is activated in metastasis and may play a role in HCC metastasis.

### Autophagy is activated in metastatic colonization of HCC

In addition to the findings that autophagy was activated in metastasis, we also found that early metastatic colony displayed higher autophagic activity than late-stage metastatic lesions. The *in vivo* GFP-LC3 analysis showed that the numbers of GFP-LC3 dots per area in small metastatic colonies (55.22±8.03) were significantly higher than those in large metastatic colonies (30.45±3.31) (*P*=0.008), which suggested higher autophagic activity in early metastatic colonies than that in late-stage metastatic lesions (Figure 4C,D). As the early metastatic colony formation represents the process of metastatic colonization, the higher level of autopahgy in early metastatic colony suggests that autophagy is activated in metastatic colonization of HCC cells.

Metastasis is a complex process involving multiple steps, including cell detachment, migration, invasion, intravasation, dissemination in circulation system, extravasation and colonization. We then examined whether autophagy altered in other steps of the metastatic cascade in addition to the metastatic colonization.

### Autophagy does not alter in cell migration and invasion

**Figure 5:**
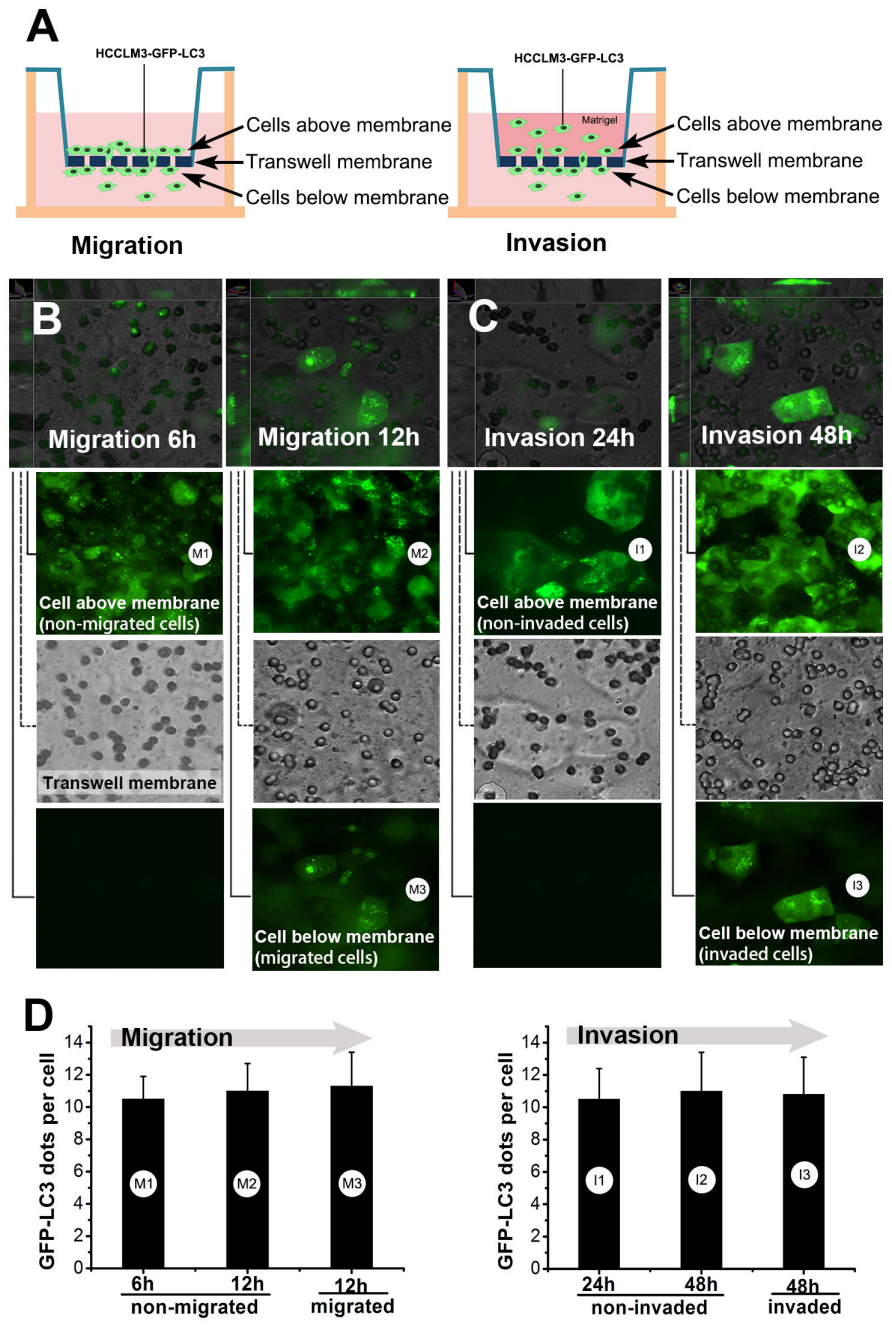
Dynamic monitoring of autophagy showed no significant changes of autophagic activity during cell migration and invasion. (A) *In* *vitro* migration and invasion models were established using stable GFP-LC3-expressing HCC cells (HCCLM3-GFP-LC3) and transwell system. (B, C) Confocal microscopic monitoring of HCCLM3-GFP-LC3 cells as they migrated through the transwell membrane or invaded through the matrigel and transwell membrane. The HCCLM3-GFP-LC3 cells migrated or invaded through transwell membrane after 6 hours and 24 hours, respectively. The images were displayed in stacked XYZ-planes mode and separated Z-plane mode. Cells above transwell membrane were non-migrated / non-invaded cells (M1, M2 / I1, I2) while cells below transwell membrane were migrated / invaded cells (M3 / I3). (D) Autophagy was quantified by counting the number of GFP-LC3 dots per cell. The numbers of GFP-LC3 dots per cell in migrated / invaded cells were not significantly different from those in non-migrated / non-invaded cells (M3 *vs*. M2, M2 *vs*. M1, M3 *vs*. M1, I3 *vs*. I2, I2 *vs*. I1, I3 *vs*. I1, all *P*>0.05).

We next examined autophagy in cell migration and invasion. *In vitro* cell migration and invasion models were established using transwell system and GFP-LC3-expressing HCCLM3 cells (Figure 5A). Dynamic monitoring of autophagy showed no significant changes of autophagic activity during invasion and migration (Figure 5B,C,D). The number of GFP-LC3 dots per cell in migrated cells (11.3±2.1 at 12h) was not significantly different from that in non-migrated cells (10.5±1.4 and 11.0±1.7 at 6h and 12h, all *P*>0.05) (Figure 5B,D). The number of GFP-LC3 dots per cell in invaded cells (10.8±2.3 at 12h) was not significantly different from that in non-invaded cells too (10.5±1.9 and 11.0±2.4 at 24h and 48h, all *P*>0.05) (Figure 5C,D).

### Autophagy does not alter in cell detachment and dissemination

**Figure 6:**
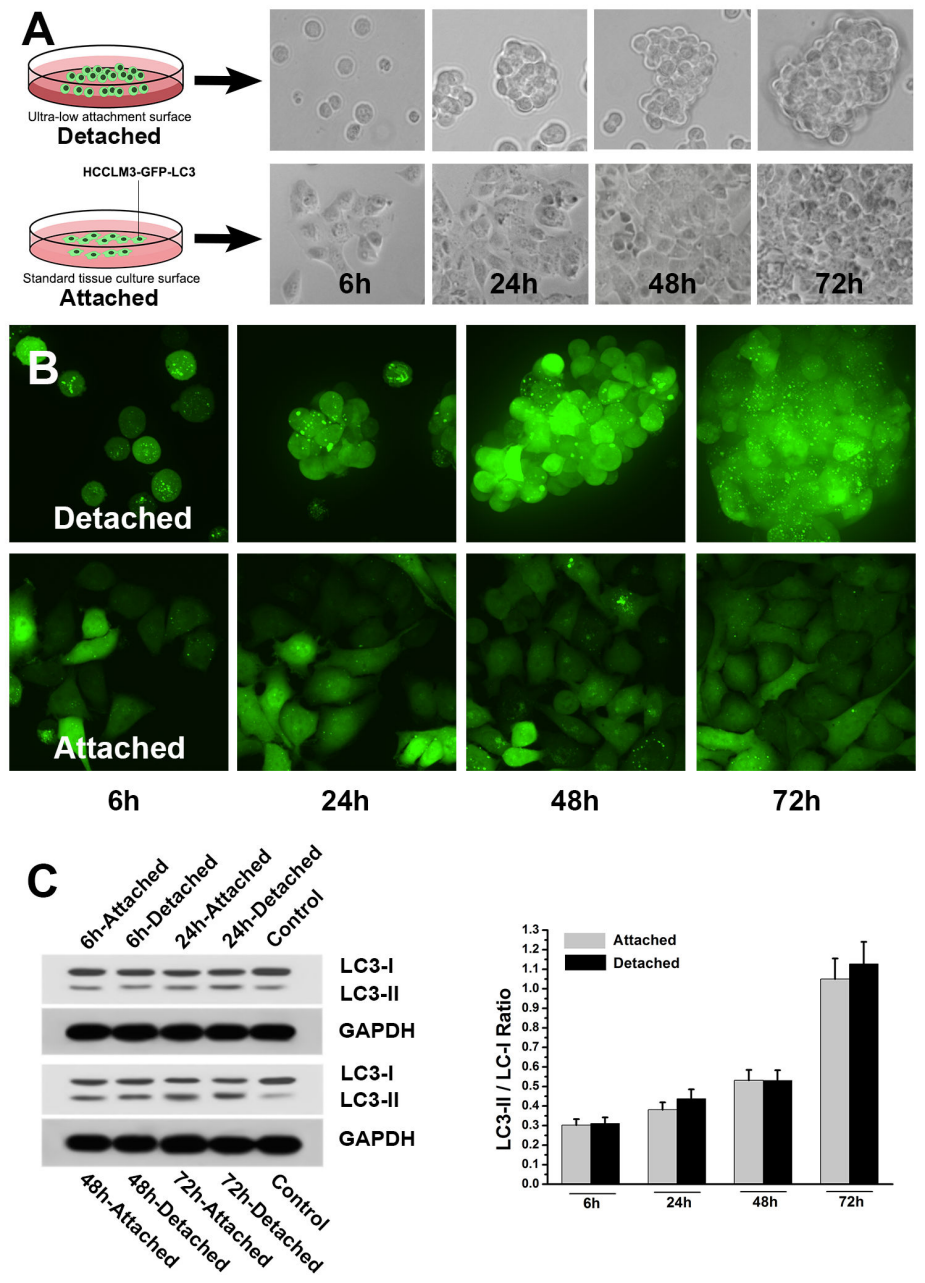
Dynamic monitoring of autophagy showed no significant autophagic alteration after cell detachment. (A) *In* *vitro* cell detachment model was established by culturing stable GFP-LC3-expressing HCC cells (HCCLM3-GFP-LC3) on dish with ultra-low attachment surface. The suspended cells maintaining in unattached state in dish with ultra-low attachment surface were detached cells, while the adhesive cells grown in dish with standard tissue culture surface were attached cells. (B) Confocal microscopic monitoring of autophagic alterations after cell detachment. (C, D) Western blot analysis showed that the LC3-II/LC3-I ratios of detached cells were not significantly different from those of attached cells at various time points (6h, 24h, 48h and 72h after cell detachment) (all *P*>0.05).

We finally examined autophagy during cell detachment. *In vitro* cell detachment and dissemination model was established by culturing HCCLM3-GFP-LC3 or HCCLM3 cells on dishes with ultra-low attachment surface (Figure 6A). Autophagic alterations after cell detachment were dynamically monitored and analyzed by GFP-LC3 analysis and western blot analysis. The dynamic monitoring showed no significant changes of autophagy after cell detachment (Figure 6B,C). The numbers of GFP-LC3 dots per cell in detached cells were 8.3±2.7, 10.9±1.9, 11.8±1.9 and 14.4±2.1 at 6h, 24h, 48h and 72h after cell detachment, which were not significantly different from those in attached cells (8.9±0.7, 10.3±1.7, 10.6±2.6 and 14.9±1.6, all *P*>0.05). Further western blot analysis confirmed the results of GFP-LC3 analysis (Figure 6C). The LC3-II/LC3-I ratios of HCCLM3 cells after detachment (detached HCCLM3 cells) were not significantly different from that of cells without detachment (attached HCCLM3 cells) at various time points (*P*=0.08, 0.08, 0.07 and 0.10 at 6h, 24h, 48h and 72h after cell detachment, respectively).

## Discussion

In this study, we examined autophagy in HCC metastasis. Our data showed that metastatic HCC cells displayed high level of autophagy, which suggested that autophagy may play a role in HCC metastasis. Our data also revealed that autophagy was activated in metastatic colonization but not in cell invasion, migration and cell detachment, suggesting that the role of autophagy may be associated with its promotion of metastatic colonization.

Detection of autophagy in metastasis (especially *in vivo*) still remains a challenge. In this study, we combined multiple methods (including LC3 immunohistochemical analysis, TEM, western blot analysis and GFP-LC3 analysis) to detect autophagy during metastasis *in vivo* and *in vitro*. The immunohistochemical analysis of autophagy using LC3 as autophagosome marker recently emerges as a valuable technique for in situ detection of autophagy. Lazova and Han et al [17-19] detected autophagy in melanoma through LC3 immunohistochemistry and found that metastatic melanoma cells displayed high level of autophagy and the upregulated autophagy was strongly associated with melanoma metastasis. Further investigations in nearly 1400 tumors from 20 types of cancer revealed that punctate LC3 expression is a common feature of malignancy and high LC3 expression was associated with invasion and metastasis [17]. The emerging evidence suggests that autophagy may be a constitutive metabolic state for metastatic cells and may play a significant role in cancer metastasis [17,19]. Our data are similar to the findings. Metastases of HCC display higher level of LC3 expression as compared with primary HCC tumors, which suggests that autophagy is activated and involved in HCC metastasis. In parallel with the LC3 immunohistochemical analysis, TEM, western blot analysis and *in vivo* GFP-LC3 analysis were also performed to compensate the potential disadvantage of the LC3 immunohistochemistry. The LC3 immunohistochemical analysis is still criticized for the reliability although it has been shown to be useful for in situ detection of autophagy. Firstly, the LC3 antibody available for immunohistochemical analysis is polycolonal antibody which can bind both LC3-I and LC3-II. Whether the upregulated LC3 expression results from the increase of LC3II (representing the level of autophagy) is uncertain. Secondly, the endogenous amount of LC3 in HCC tissues is shown to be low. In the context of low LC3 expression analysis using LC3 as autophagic marker is error-prone. Martinet et al [21] reported that LC3 appears to be valuable for detecting autophagy only when it is overexpressed (e.g., GFP-LC3 overexpression) [21,22]. To avoid misinterpretation, TEM, GFP-LC3 analysis and western blot analysis are often used to validate the findings. So far TEM still remains the gold standard technique for in situ detection of autophagy. The TEM analysis in this study confirmed the findings from LC3 immunohistochemistry. However, it was shown to be too labor-intensive and not suitable for quantitative analysis of autophagy as described [21]. Moreover, neither TEM nor LC3 immunohistochemistry lends itself well to dynamic observation [23]. Therefore, we applied GFP-LC3 analysis to detect autophagy in metastasis. The GFP-LC3 analysis has been applied to whole animals (e.g., GFP-LC3 transgenic mice) and has been demonstrated to be particularly useful in monitoring autophagy *in vivo* [23,28,29]. In this study, we established a highly metastatic HCC cell line stably expressing GFP-LC3 reporter (HCCLM3-GFP-LC3) and used the HCCLM3-GFP-LC3 cells to develop mouse model of pulmonary metastasis and *in vitro* cell migration, invasion and detachment models. The new models enable us to trace autophagy in metastasis and analyze the possible role of autophagy in individual steps of the metastatic cascade. For the first time, we have a deeper look at autophagy in metastases and dynamically monitor the autophagic alteration in cell migration, invasion and detachment. The GFP-LC3 analysis not only confirmed the findings from LC3 immunochemistry and TEM but also showed that autophagy was activated in metastatic colonization but not in invasion, migration and detachment. The results suggested that autophagy may play a role in metastasis (especially in metastatic colonization of HCC cells). Autophagy may be activated to overcome metabolic stress and maintain viability of HCC cells to promote metastatic colonization. Our data showed that autophagy appeared to be not involved in cell migration, invasion and detachment. This was contrary to the expectation. It is speculated that autophagy may be activated during these processes, especially in cell detachment. It is reported that cell detachment can strongly induce autophagy in mammary epithelial cells detached from extracellular matrix (ECM), which suggested that autophagy may play a role in anoikis resistance, cancer dissemination and metastasis [16]. Similarly, cell detachment may also induce autophagy in metastatic HCC cell. However, our data showed that HCC cells detachment did not induce autophagy. This may be due to the different study model (3D-model vs. 2D-model) and cell lines (mammary epithelial cells vs. HCC cells). Analysis using a similar model or preferably in an *in vivo* model is expected. Autophagy *in vivo* is considered to be highly complex and quite different from that *in vitro*. How to dynamically monitor autophagy *in vivo* remains a challenge. The combination of zebrafish model of HCC metastasis and GFP-LC3 analysis may overcome the problem. The zebrafish is optical transparent and xenograft transplantable, which provides an ideal organism for *in vivo* analysis of autophagy in metastasis [30-33]. Meanwhile, our study shows that the stable GFP-LC3-expressing HCC cells are valuable for monitoring autophagy *in vivo*. If we could established a zebrafish model of HCC metastasis using GFP-LC3 expressing HCC cells, we would be able to dynamically monitor autophagy *in vivo* and determine autophagic alterations during the whole process of metastasis. Therefore, establishing the zebrafish model of HCC metastasis using GFP-LC3 expressing HCC cells is now underway in our institute. With aid of the new model, the dynamic *in vivo* analysis of autophagy in the entire process of HCC metastasis is expected to be presented in the future.

In conclusion, this study shows that autophagy is upregulated in HCC metastasis and it is activated in metastatic colonization but not in cell invasion, migration or detachment. Our data suggest that autophagy may play a role in HCC metastasis through promoting metastatic colonization of HCC cells.

## Supporting Information

Figure S1 **Western blot analysis of LC3 and p62 in paired primary tumors and metastases (intravascular metastasis, intrabiliary metastases, bone metastases, lymph node metastases and lung metastases).** The LC3-II/LC3-I ratios of HCC cells in metastases were significantly higher than those in primary tumors while the p62 levels of HCC cells in metastases were remarkably lower than those in primary tumors. The relative LC3-II/LC3-I ratios and the relative p62 levels were displayed (*P<0.05).
(DOCX)
