# Supplementary figures and images for: Promoting Colonization in Metastatic HCC Cells by Modulation of Autophagy
Source: PLoS One. 2013 Sep 13;8(9):e74407. doi: 10.1371/journal.pone.0074407 (PMC3772859; doi:10.1371/journal.pone.0074407)

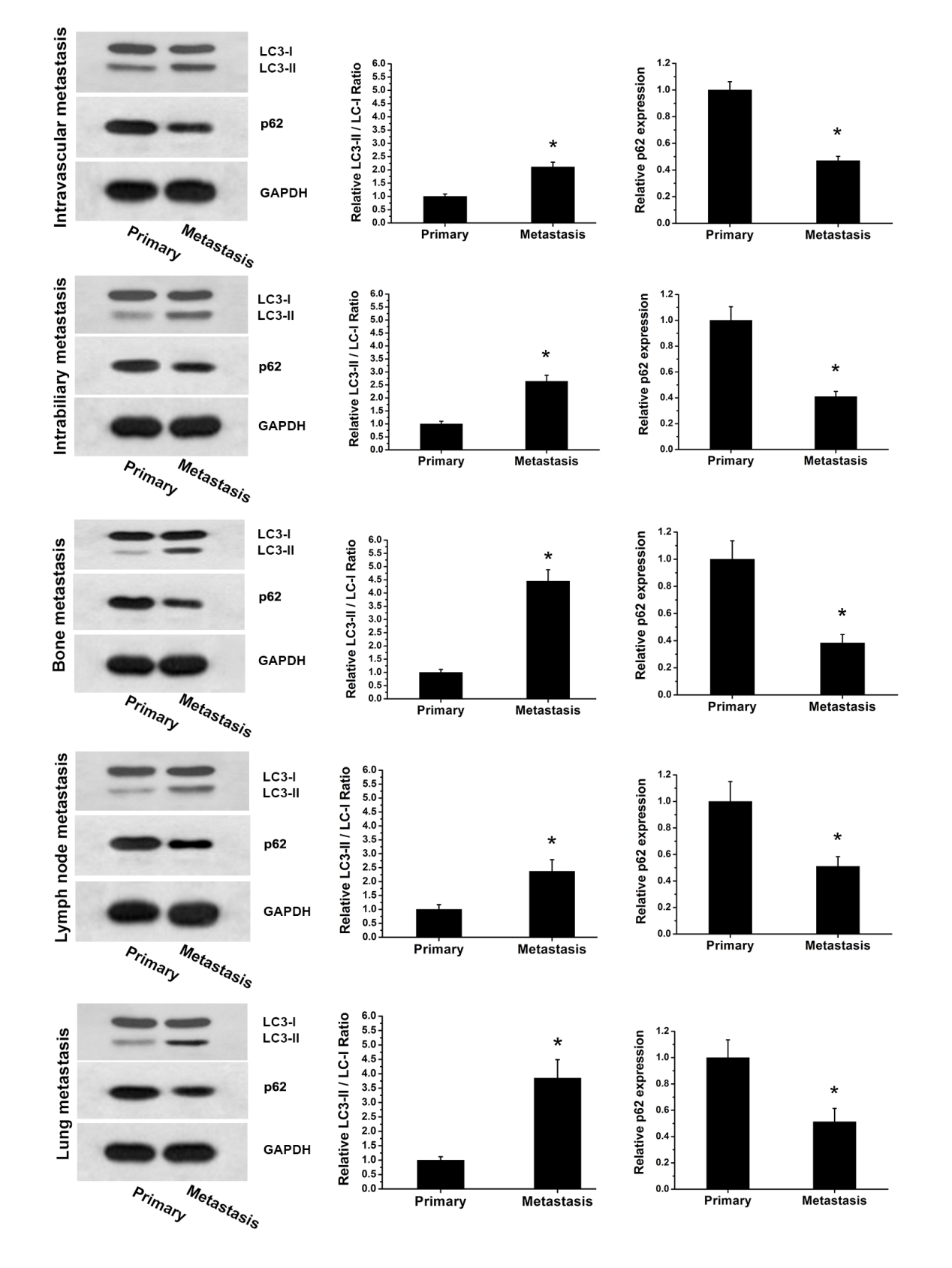

Supplement: Figure S1 — Western blot analysis of LC3 and p62 in paired primary tumors and metastases (intravascular metastasis, intrabiliary metastases, bone metastases, lymph node metastases and lung metastases). The LC3-II/LC3-I ratios of HCC cells in metastases were significantly higher than those in primary tumors while the p62 levels of HCC cells in metastases were remarkably lower than those in primary tumors. The relative LC3-II/LC3-I ratios and the relative p62 levels were displayed (*P<0.05). (DOCX) [file pone.0074407.s001.docx]
